# Heterotrimeric G proteins as emerging targets for network based therapy in cancer: *End of a long futile campaign striking heads of a Hydra*

**DOI:** 10.18632/aging.100781

**Published:** 2015-07-20

**Authors:** Pradipta Ghosh

**Affiliations:** Department of Medicine, University of California at San Diego, La Jolla, CA 92093, USA

**Keywords:** GIV, Girdin, growth factor receptor tyrosine kinases, cancer metastasis, G protein -coupled receptors, heterotrimeric G proteins

## Abstract

Most common diseases, e.g., cancer are driven by not one, but multiple cell surface receptors that trigger and sustain a pathologic signaling network. The largest fraction of therapeutic agents that target individual receptors/pathways eventually fail due to the emergence of compensatory mechanisms that reestablish the pathologic network. Recently, a rapidly emerging paradigm has revealed GIV/Girdin as a central platform for receptor cross-talk which integrates signals downstream of a myriad of cell surface receptors, and modulates several key pathways within downstream signaling network, all via non-canonical activation of trimeric G proteins. Unlike canonical signal transduction via G proteins, which is spatially and temporally restricted, the temporal and spatial features of non-canonical activation of G protein via GIV is unusually unrestricted. Consequently, the GIV●G protein interface serves as a central hub allowing for control over several pathways within the pathologic signaling network, all at once. The relevance of this new paradigm in cancer and other disease states and the pros and cons of targeting the GIV●G protein interface are discussed.

Cancer, like most other chronic diseases, is a signal transduction disease par excellence, a consequence of aberrant transmission of environmental cues to the interior of the cell via a complex network of signaling hubs. Heterotrimeric G proteins are one such major signaling hub that are essential components of the signal gating machinery in healthy eukaryotic cells. They serve as molecular switches for signal transmission via 7 transmembrane domain G protein coupled receptors (GPCRs) to intracellular effectors [39]. Activation of G proteins is tightly regulated by a network of modulators: guanine nucleotide exchange factors (GEFs) trigger activation, GTPase activating proteins (GAPs) enhance inactivation, and finally, guanidine dissociation inhibitors (GDIs) uncouple the trimer and maintain the G protein in an inactive (GDP-bound) conformation [40]. These modulators function coordinately to maintain finiteness of signal transduction via G proteins [41], mostly by ensuring that activation of G proteins is spatially and temporally restricted, i.e., triggered exclusively at the plasma membrane (PM) by agonist activation of GPCRs via a process that is completed within a few hundred *milliseconds* [42].

The importance of maintaining the critical balance between G protein activation and deactivation and the loss of such balance in cancer has been highlighted by studies on several cancer-associated mutants of trimeric G protein α-subunits and GPCRs (reviewed in [43, 44]). These mutations trigger malignant transformation and oncogenesis by rendering the G proteins constitutively active in the GTP-bound conformation either by impairing its intrinsic ability to hydrolyze GTP (i.e., GTPase-deficient) or by reducing its sensitivity to the action of GAPs (i.e., GAP-insensitive). Thus, it has now been established that “*hyperactivation of G proteins*” is a *bona-fide* basis for oncogenic signaling via trimeric G proteins.

Despite the insights gained, the rare oncogenic driver mutations in G proteins in a handful of cancers do not explain the basis for deregulated G protein signaling in the vast majority of cancers that do not harbor mutant G or GPCR proteins. A growing body of work by us and others [24, 45, 46] have indicated that genetic or epigenetic factors that deregulate the intricate network of G protein regulatory proteins are just as significant as those that directly affect the G proteins /GPCRs, if not more. More specifically, a recently identified family of non-receptor GEFs, called rheostats [35] best exemplify the wide prevalence and broad significance of deregulated G protein regulatory network in cancers. Rheostats like GIV (Gα-Interacting Vesicle-associated; a.k.a Girdin) [24] and other members of this family, are non-receptor GEFs for trimeric G proteins; they derive their name based on their ability to 'adjust' the duration of G protein signaling depending on the abundance of functional copies of the GEF in cells [35]. Studies on GIV-GEF have led to the rapid emergence of a new paradigm in non-canonical activation of trimeric G proteins that has distinctive temporal and spatial features. Such activation appears to be less constrained and less restricted than canonical G protein activation by receptor GEFs (i.e., GPCRs) in three major ways (summarized in [24]): 1) G proteins can be transactivated by diverse classes of receptors, e.g., growth factor RTKs, TLRs, integrins and GPCRs--many of which are typically not known to bind or activate G proteins; 2) G proteins both at the PM and on internal membranes that are discontinuous with the PM can be activated; and 3) Activation continues for prolonged periods of time (as opposed to milliseconds). While the molecular mechanisms that govern such non-canonical G protein activation and the variety of pathways it modulates (summarized in Figure 1) are still unfolding, the relevance of this new paradigm in cancer and other diseases is clear (summarized in [24]).

**Figure 1:**
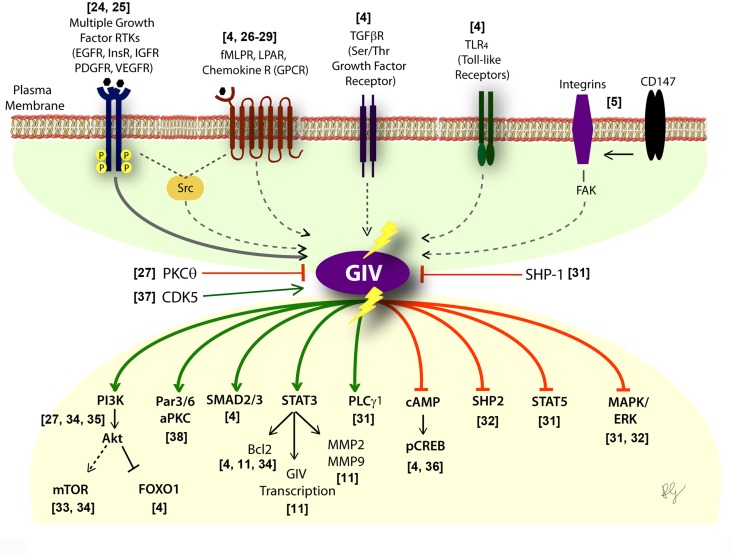
Activation of G proteins by GIV-GEF modulates multi-receptor signaling and broadly impacts the downstream signaling network Schematic showing the diverse classes of receptors (upper half) which sense a variety of chemical signals, that converge on GIV. Lower part shows the consequence of non-canonical transactivation of G proteins by GIV (when GIV-GEF is functionally intact or turned "ON") on the multitude of downstream pathways within the signaling network. Green = enhancement; Red = suppression. Shown in the middle are three known ways to either inhibit (PKCθ selectively phosphoinhibits GIV-GEF [27]; SHP-1 dephosphorylates tyrosine-phosphorylated GIV [30]) or activate (CDK5 phosphoactivates GIV-GEF [37]) GIV-dependent signaling.

Most of these diseases, if not all, are characterized by cellular processes (migration, proliferation, apoptosis/survival, autophagy, secretion, etc) that are driven by more than one receptor or one class of receptors, and most often require synergistic signaling of diverse classes of receptors. GIV appears to serve as a platform on which crosstalk between diverse classes of receptors either directly (in the case of RTKs [24]) or indirectly (via mechanisms that are still unclear) converge; GIV's intrinsic GEF activity subsequently translates the converging signals into activation of Gαi in the vicinity of activated receptors. The impact of such transactivation on downstream signals is equally diverse (Figure 1). When GIV-GEF is transcriptionally upregulated [11, 38] and/or turned "ON" by phospho-activation [37], Gαi is activated and multiple signaling pathways are either enhanced or suppressed, thereby affecting an entire network, not just individual pathways. Conversely, when GIV-GEF is turned "OFF" by selective phosphoinhibition [27] or alternative splicing [31, 35], Gαi activation cannot be coupled to incoming signals; consequently, the network assumes a yin-yang mirror image pattern (Figure 1).

Given the broad landscape of signaling pathways that GIV modulates, and its ubiquitous nature of expression, it is not surprising that deregulation of GIV-GEF drives several pathophysiologic conditions (Table). In the context of cancer, it is clear that high copies of GIV means unrestricted G protein signaling and propagation of signals that enhance tumorigenesis (like invasiveness, stemness and chemoresistance; [24], Table) regardless of the receptor of origin. Given the nature of receptor classes modulates and the prometastatic nature of the signaling pathways enhanced, GIV's expression at high levels carries a poor prognosis across a broad range of solid tumors (Figure 2). Although prometastatic signaling is the most well understood role of GIV, the striking yin-yang effect of GEF-ON *versus* GEF-OFF states has also been described in the context of some other cellular processes and diseases, e.g., fibrosis, wound healing, diabetes [24], most, if not all these diseases are also multi-receptor in origin (Table; green columns). It is possible that a similar yin-yang effect modulates all other diseases where GIV's role has been defined but the role of its GEF function has not (Table; red columns).

**Table:** 

Disease/Pathology Investigated		Effect of GIV's GEF function	Receptor(s) Studied	Citation
**Cancer Progression**	Migration/Invasion	“ON” = Enhances “OFF” = Inhibits	IGF1R, EGFR, Multi-receptor*	[8, 11, 36, 51, 52]
	Stemness	Not examined	--	[19]
	Chemoresistance	Not examined	--	[53]
	Tumor-Stroma Interactions	Not examined	PDGFR, TGFβR, CXCR4	[2]
	Tumor angiogenesis	Not examined	VEGFR	[54]
**Organ Fibrosis (Liver)**	Myofibroblast transdifferentiation, collagen production, chemotaxis, mitosis, anti-apoptotic signaling	“ON” = Enhances “OFF” = Inhibits	PDGFR, CCR1, TGFβR	[4]
**Dermal Wound Healing**	Wound closure	“ON” = Enhances “OFF” = Inhibits	Multi-receptor*	[52]
**Nephrotic Syndrome**	Podocyte survival after glomerular injury	“ON” = Enhances survival “OFF” = Inhibits survival	VEGFR	[34]
**Insulin Resistance, Type II Diabetes**	Metabolic insulin response in the skeletal muscle	Not examined	InsR	[55]
**Disorders of Blood Vessels**	Neonatal vascular development; Pathologic neovascularization; vein repair; vein graft	Not examined	PDGF, Angiotensin II, VEGF	[56-59]
**Neuronal Plasticity, Memory formation**	Synaptic plasticity	Not examined	NMDA	[60]

**Figure 2:**
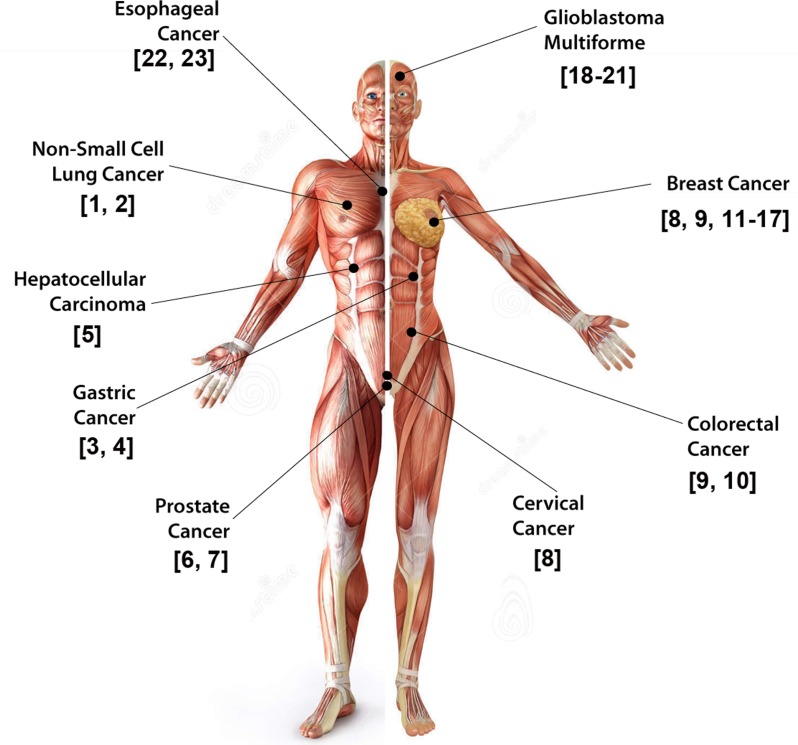
GIV is a bona-fide prometastatic protein Schematic summarizing the variety of solid tumors in which elevated expression of GIV/Girdin in tumor cells has been linked to its role in imparting stemness, invasiveness, prometastatic and anti-apoptotic signaling, aggressiveness and poor clinical outcome has been studied.

Finally, the ability to manipulate a broad signaling network and reset it from an unstable pathologic to a stable physiologic state downstream of multiple receptors by fine-tuning GIV's GEF function is an attractive concept because of many reasons: 1) Eliminates the need to block physiologic signaling via cell surface receptors; 2) Overcomes the limitations of unknown upstream and downstream components; 3) Preserves the utility of biomarkers/therapeutic targets despite re-wiring of signaling pathways during the course of disease progression. Recently, in a proof-of-concept study [47] using cell permeable peptides exogenous modulation of GIV's GEF function allowed resetting pathologic signaling networks and phenotypes in diverse cell types, while sparing individual receptors. Such a strategy represents a fundamental deviation from the current strategy of individual pathway/receptor-blockade, that sooner or later fails due to a switch in addiction of the tumor from the targeted pathway to other pathways [48]. An appropriate analogy for such individual receptor/pathway blockade is the futility of severing the heads of a Hydra; for each head severed, two more grows in its place. While the studies on GIV-GEF indicate that it may be an unusual hub for convergent multi-receptor signaling for the broad modulation of the "disease network", and raise our hope that the GIV●Gαi-interface may serve as an effective target for therapy, several hurdles lie ahead of us before such a possibility can be realized. For example, targeting a protein like GIV, which is expressed ubiquitously and serves a long list of roles in normal tissues [24] may carry an insurmountable risk of side effects. Even if targeting the targeted therapy selectively to the tumor cells overcomes the first challenge, the second challenge is that inhibition of the GIV●Gαi-interface may inadvertently disrupt also signaling via other members of this family [49, 50] that share a similar structural basis for activating G proteins.

In conclusion, through the studies on GIV, we have obtained a sneak preview of just how large the footprint of oncogenic signaling via trimeric G proteins could be. Because GIV is just one of the members of the rheostat family, we are likely seeing only the proverbial tip of the iceberg, and a lot more must be known before any of these findings can be translated to transformative and impactful therapies and/or biomarkers for personalized care.
